# Practicing Digital Gastroenterology through Phonoenterography Leveraging Artificial Intelligence: Future Perspectives Using Microwave Systems

**DOI:** 10.3390/s23042302

**Published:** 2023-02-18

**Authors:** Renisha Redij, Avneet Kaur, Pratyusha Muddaloor, Arshia K. Sethi, Keirthana Aedma, Anjali Rajagopal, Keerthy Gopalakrishnan, Ashima Yadav, Devanshi N. Damani, Victor G. Chedid, Xiao Jing Wang, Christopher A. Aakre, Alexander J. Ryu, Shivaram P. Arunachalam

**Affiliations:** 1 GIH Artificial Intelligence Laboratory (GAIL), Division of Gastroenterology and Hepatology, Department of Medicine, Mayo Clinic, Rochester, MN 55905, USA; 2 Microwave Engineering and Imaging Laboratory (MEIL), Division of Gastroenterology and Hepatology, Department of Medicine, Mayo Clinic, Rochester, MN 55905, USA; 3 Division of Gastroenterology and Hepatology, Mayo Clinic, Rochester, MN 55905, USA; 4 Department of Medicine, Mayo Clinic, Rochester, MN 55905, USA; 5 Department of Cardiovascular Medicine, Mayo Clinic, Rochester, MN 55905, USA; 6 Department of Internal Medicine, Texas Tech University Health Science Center, El Paso, TX 79995, USA; 7 Department of Radiology, Mayo Clinic, Rochester, MN 55905, USA

**Keywords:** phonoenterogram, PEG, computer-aided auscultation, bowel sounds, artificial intelligence, microwave telemetry, microwave acoustic sensors, gastroenterology, digital health

## Abstract

Production of bowel sounds, established in the 1900s, has limited application in existing patient-care regimes and diagnostic modalities. We review the physiology of bowel sound production, the developments in recording technologies and the clinical application in various scenarios, to understand the potential of a bowel sound recording and analysis device—the phonoenterogram in future gastroenterological practice. Bowel sound production depends on but is not entirely limited to the type of food consumed, amount of air ingested and the type of intestinal contractions. Recording technologies for extraction and analysis of these include the wavelet-based filtering, autoregressive moving average model, multivariate empirical mode decompression, radial basis function network, two-dimensional positional mapping, neural network model and acoustic biosensor technique. Prior studies evaluate the application of bowel sounds in conditions such as intestinal obstruction, acute appendicitis, large bowel disorders such as inflammatory bowel disease and bowel polyps, ascites, post-operative ileus, sepsis, irritable bowel syndrome, diabetes mellitus, neurodegenerative disorders such as Parkinson’s disease and neonatal conditions such as hypertrophic pyloric stenosis. Recording and analysis of bowel sounds using artificial intelligence is crucial for creating an accessible, inexpensive and safe device with a broad range of clinical applications. Microwave-based digital phonoenterography has huge potential for impacting GI practice and patient care.

## 1. Introduction

Gastrointestinal diseases have significant implications on morbidity, mortality and quality of life in affected individuals. For instance, functional gastrointestinal diseases that produce symptoms, without any structural or visible pathological lesions, affect more than 40% people worldwide, according to a large multinational survey [1]. Irritable bowel syndrome (IBS) is a chronic functional disorder [2] that is diagnosed after excluding other medical conditions and fulfilling a clinical criterion. The lack of a definite test for IBS makes it a challenge for both physicians and patients, with the latter often undergoing extensive testing to rule out medical conditions, leading to higher expenditures and lower quality of life [3,4].

Similarly, managerial gaps exist in conditions such as post-operative ileus (POI) and intestinal obstruction. POI refers to disruption of normal bowel motility following surgery, leading to obstipation and intolerance to oral intake [5]. Physicians typically rely on clinical signs such as passage of flatus and intestinal auscultation to decide on the time to start oral feeds [6]. However, this method may not be reliable indicator as it is difficult to determine in unconscious patients and patients with prolonged POI and depends on subjective interpretation of clinical signs and clinical experience [7]. Imaging modalities can be used in these cases, but they increase radiation exposure and have limitations subject to availability. In emergent conditions such as intestinal obstruction, guidelines suggest plain X-ray or an abdominal CT scan for diagnostic confirmation [8]. While this is a reliable method, it presents time constrains for patients with unstable vitals who are often taken directly into surgery without any preliminary testing.

Furthermore, in chronic conditions such as ulcerative colitis (UC) and Crohn’s disease (CD), frequent longitudinal monitoring with endoscopy is required to track severity and guide management protocols [9]. With the number of endoscopies increasing every year and countries such as the United States reporting 22.2 million endoscopies in 2021 [10], there is an increased burden on the healthcare system. Invasive procedures such as endoscopies increase the risk of infection and perforation in individuals [11]. In addition, they are costly, require substantial healthcare personnel, and are not a feasible option in resource limited settings. Therefore, there is an urgent need for alternative diagnostic modalities that relieve pressure on the healthcare system, reduce the number of invasive procedures on patients requiring frequent monitoring, are safe, cost-effective, and easily accessible and available, which can help in timely diagnosis and guide management.

In the search for a solution to the existing problems, there has been increasing research in the recent past to utilize bowel sounds (BS) as a new diagnostic tool. However, intestinal auscultation, which once drew considerable interest [12,13,14], is sparsely used in clinical practice today due to a lack of standardized recording technologies, interpersonal variations in interpretations, and poor understanding of the underlying physiology and clinical applications [15]. The term ‘phonoenterography’ was coined by Watson and Knox [16] in 1967 to describe the recording and analysis of BS. In the recent past, significant improvements have been made in developing a recording device that accurately detects and defines BS while differentiating it from other acoustic signals from the body. Moreover, there has been significant research in developing computer aided auscultation (CAA) [17,18] to reduce the interpersonal variability and subjective bias. Several review papers [19,20] have summarized the advancements in recording technologies with the latest studies using wireless devices [21,22] to record and transmit data. Analysis of BS can be used for diagnosis and or management of common gastrointestinal conditions such as intestinal obstruction [23], acute appendicitis [24], inflammatory bowel disease [25], diverticular disease [25], bowel polyps [25], ascites [26], post-operative complications and critical care [27] and irritable bowel syndrome [17]. It has also used in management of diabetes mellitus [28,29], neurodegenerative disorders [30] and the diagnosis of infantile hypertrophic pyloric stenosis [31].

While existing literature highlights the need for this technology, it fails to provide a clear understanding of the mechanism of BS production, its clinical usability, and the future of using a digital BS detector using a microwave-based sensor for recording phonoenterogram (PEG). The purpose of this review was to study the physiology of BS origin, factors affecting its frequency, clinical applications and recording technologies in existing literature. Additionally, this review reflects on the prospects of using microwave-based systems for PEG and its impact on transforming gastroenterological practice for improving patient care.

## 2. Physiology

**Figure 1:**
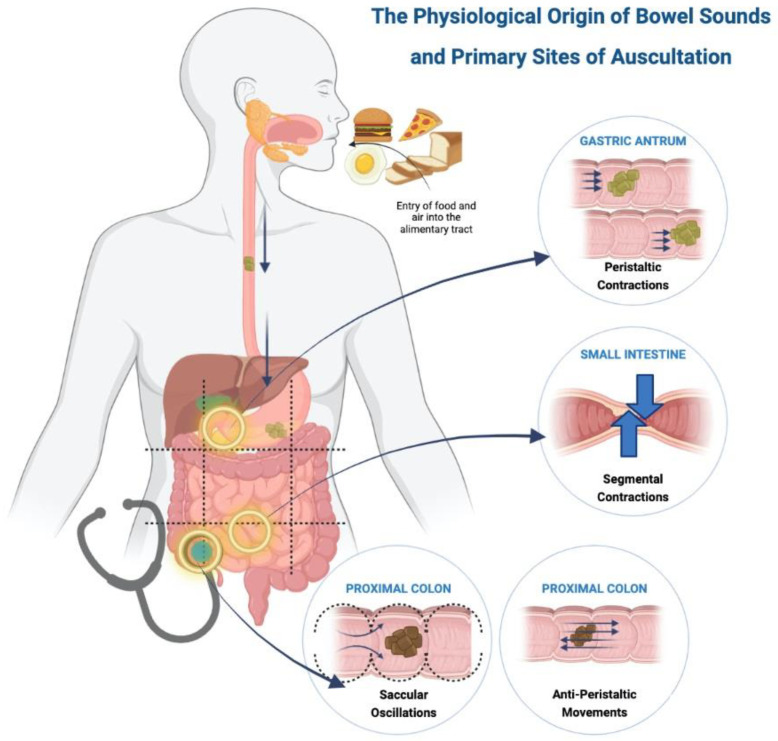
Physiological origin of bowel sounds and primary sites of auscultation.

The physiology of bowel sounds dates back to early 1900s where a detained explanation was given by Cannon [12], Plessis [13] and Milton [14]. Currently, we do not have an exact mechanism for the production of bowel sounds, but a majority suggest that intestinal motility is the primary origin [12,13,14]. Gut motility, contents of the gastrointestinal lumen and the presence of gas have been hypothesized as the major contributing factors [32,33,34,35]. Air that is consumed with food reaches the lumen of the gut, where gut motility leads to constant formation and resolution of gas bubbles [33] that generate sound in various portions of the gastrointestinal tract [36] (Figure 1) [37].

### 2.1. Gastric and Pyloroduodenal Region

Food reaches the stomach and is pushed forward via peristaltic movements towards the pylorus [1]. The frequency of gastric peristalsis and pyloric sphincter relaxation do not coincide, leading to food hitting against the closed sphincter, which produces a loud, explosive sound described as ‘bursting of bubbles’ [12]. Peristaltic waves occur around 3 times per minute or every 20 s as cited by Plessis [13] and confirmed by Moritz’s experiment on himself [12]. These propulsive movements are normally painless but can produce pain with an exaggerated sound in intestinal obstruction [13].

### 2.2. Small Intestine

The bolus of food in the small intestine is broken down into smaller fragments by segmental contractions of the circular muscles that occur about seven to twelve times a minute [13]. These contractions push the food forward and backward to allow mixing of the food with the intestinal secretions. Thus, a large number of contractions are required in the small intestine to propel the food forward. Additionally, intestinal motility is affected by bowel tone [12], creating a pressure gradient with higher tone in the upper gut as compared to the lower, aiding in downward movement of the food. Bowel sounds arising from the small intestinal have three distinctive features, namely [12]: (i) Pattern—slowly rising and gradually subsiding, or slowly rising with a peak and sudden drop, or sharply rising and gradually dropping; (ii) Rhythm—each bowel sound lasts for two to three seconds with multiple sounds occurring in same location for several minutes; and (iii) Intensity—loud sounds due to the presence of *valvulae conniventes* that alter the luminal diameter and contribute to pressure changes.

### 2.3. Ileocecal Region and Colon

Movements in the proximal colon are explained by two theories, namely anti-peristalsis and saccular oscillations. The food moving from the ileum to the caecum acts as a stimulus causing the caecum to contract and form a blind pouch which temporarily prevents the progression of food, creating a high-pressure zone. Food is pushed back towards the caecum due to this pressure gradient, and it strikes the ileocecal valve, thus producing a sound. This phenomenon is called anti-peristalsis [12,14]. The colon has numerous sacculae which produce oscillatory movements with the intestinal contents and contract to push the contents into the next sacculi. This phenomenon allows churning of the food and produces a sound described as a continuous popping and gurgling noise. Some researchers [12,38] believe the saccular oscillations contribute more to the bowel sound production than antiperistalsis. The right lower quadrant is a point of auscultation due to more activity in the ileocecal and proximal colon, as compared to the distal colon. Contractions from the distal colon push the contents forward and produce crackling noises followed by an urge to pass flatus [12].

Although bowel sound production and intestinal motility have been closely linked, there are studies that contradict this theory as bowel sounds have been recorded in abdominal quadrants independent of peristalsis, indicating they may not be a combined event [39]. Tomomasa et al. [40] suggested bowel sounds are a result of the transfer of energy between the contents of the lumen rather than propulsion. This phenomenon occurs during the second phase of migrating motor complex (MMC) in a fasting state. MMC refers to the motor activity of the intestine with three phases, namely quiescent motor, irregular and regular pattern of contractions [41]. Another study [42] suggested myoelectrical slow wave and spike burst activity of the intestine as the etiology leading to bowel sound production. Dual peaks of bowel sounds are heard after consumption of food [32,43,44]. The first occurs immediately after the meal and is hypothesized to be due to swallowed air forming intraluminal gas. The second occurs an hour later, which coincides with gastric emptying. The stomach is the most active site of bowel sound production, followed by the colon and then the small bowel [34]. Short frequency high amplitude sounds are produced in the colon whereas higher frequency sounds originate from the stomach. Sometimes a loud rumble [45] can be heard from the abdomen, which can be due to a pathological cause such as gut hypertrophy or due to physiological nervous air swallowing.

## 3. Effect of Modifiable and Non-Modifiable Factors on Bowel Sounds

Studies have researched the effect of various modifiable and non-modifiable factors on bowel sounds. Knowledge pertaining to these factors can help propagate further research in the following scenarios:

### 3.1. Serum 5-Hydroxytryptamine

Serum 5-Hydroxytryptamine (5-HT) is produced by the intestine in response to pressure changes and intestinal epithelium deformation. Increased bowel motility leads to increased release of 5-HT into the blood, producing intestinal symptoms in carcinoid syndrome [46]. 5-HT thus acts as a local hormone causing excessive loud bowel sounds known as borborygmi [46].

### 3.2. Medications

Tomomasa et al. [40] studied the relationship between gastrointestinal sounds and small intestinal motility. Their results concluded that the sum of sound index (SI) coincides with the gastric phase of migrating motor complex, with a lower SI seen in somatostatin [31,40] and scopolamine (due to decreased antral contraction and delayed gastric time respectively). A higher SI is seen with erythromycin and metoclopramide (due to increased antral contraction and shorter transit time, respectively). Gut stimulants such as carbachol and magnesium sulphate lead to an increased production of bowel sounds [46]. Furthermore, Martin et al. [47] studied the effect of anti-spasmodic drugs, oxybutynin and dicyclomine on gastrointestinal activity using a microphone with a panasonic recorder embedded in a polystyrene cotton-padded box. A decrease in bowel sounds following drug administration was noted. Another study by Emoto et al. [48], using autoregressive moving average (ARMA) spectrum to study the effect of mosapride, found a decreased sound to sound interval with increasing plasma concentrations of mosapride and peak gut activity. They concluded that this technique was highly sensitive and specific to detect bowel sounds.

### 3.3. Morphine

The post-operative course of a patient is determined by the status of bowel function and tolerability of feeds. Morphine and meperidine, used for postoperative pain control, decrease gut motility by inhibiting myoelectric complexes in the small intestine and colon [49]. A positive correlation [50] between the quantity of morphine used and the time of the return of bowel sounds, first flatus, and first bowel movement was found. However, there was no correlation between incision length and bowel motility. Limited use of morphine is recommended to attain early return of bowel function [50].

### 3.4. Coffee and Soda

Recreational drinks such as coffee and soda can be used for the treatment of constipation [51]. Coffee produces gastrin hormone in the pyloric antrum, whereas the carbon dioxide in the soda produces intraluminal gas that creates pressure in the gastrointestinal tract leading to increased gut motility [51,52]. Additionally, soda excites the trigeminal neurons in the tongue that stimulates the dorsal vagus nucleus in the brainstem, further activating the visceral sensory neurons to promote gut motility [53].

### 3.5. Stress

Holtmann and Enck noted that physical and physiological stressors lead to increased non-propulsive contractions of the esophagus, decreased antral motility of the stomach, decreased migrating motor complexes in the small intestine and an increased motor spike activity in the colon [54].

### 3.6. Age and Gender

Gastrointestinal motility is affected by non-modmodifiable factors such as age and gender. Safronov et al. [18] used computerised phonoenterography(CPEG) to study various sound indices (amplitude, frequency and duration) in different age groups and recorded the peristaltic sounds as gastric images. High fasting CPEG indices were seen in those between 6–9 years, whereas weak post-meal bowel motility and low motor evacuation was seen in ages 6–15 years. However, no significant difference between gender was seen.

## 4. Clinical Application of Bowel Sounds

**Figure 2:**
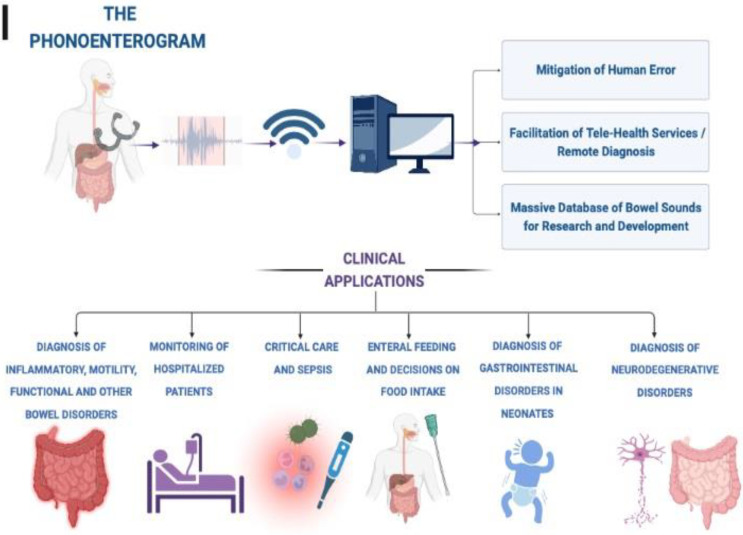
Potential clinical applications of digital phonoenterography.

Recording and analysis of bowel sounds using a phonoenterogram can function as a diagnostic modality and aid in the management of various clinical conditions (Figure 2) [37]. Some clinical scenarios for application of bowel sounds are described below:

### 4.1. Intestinal Obstruction

Intestinal obstruction requires timely diagnosis for emergent management strategies [8,55]. Bowel sounds are initially heard as “tinkling” high pitched sounds, followed by muffled or absent sounds as the obstruction progresses [56]. Ching et al. [23] indicated the use of bowel sound features to localize the site of obstruction. Higher peristalsis with a large volume shift and competent ileocecal valves causes a higher sound frequency and duration in the large bowel as compared to small bowel obstruction. A longer sound-to-sound interval (SSI) was noted in surgery, requiring comparison to the non-surgery requiring small bowel obstruction patients. However, no significant difference in the SSI, dominant frequency and the peak frequency among cases of acute, sub-acute, and no bowel obstruction was observed. Yoshino et al. [57] found patients with intestinal obstruction to have a higher frequency histogram compared to healthy controls. Bowel sound auscultation was found to have a high sensitivity and specificity with a high rater agreement [58].

### 4.2. Acute Appendicitis

Local inflammation around the appendix influences peristalsis, resulting in changes of bowel sound character [59,60]. The literature suggests that one-fourth of the patients undergoing appendicectomy have a normal appendix at operation [24]. Abdominal auscultation to analyze bowel sound features can aid in diagnosis to prevent unnecessary abdominal surgeries in such patients. Arnbjörnsson et al. [24] recorded bowel sounds pre and post appendicectomy in clinically diagnosed patients and found patients with gangrenous appendix to have a significant difference in pre- and post-operative median height of spike frequency, whereas patients with normal appendix had no significant difference in the two groups. Furthermore, this study stressed the importance of repeated recordings to avoid variations produced by abdominal movements such as breathing or muscle contractions which tend to affect the amplitude and frequency of bowel sound recording.

### 4.3. Large Bowel Disorders

Bowel sound features have been studied in pathologies of the large intestine such as Crohn’s disease, ulcerative colitis, diverticular disease, and bowel polyps [61]. Hadjileontiadis et al. [61] studied scatter plots of higher order crossings, and found an overlap between post-polypectomy patients and healthy subjects, suggesting bowel sounds as a potential scale to determine the efficacy of the surgical procedure. Inflammatory bowel disease (IBD) is a chronic condition requiring repeated testing using colonoscopies [9], thus the need for a non-invasive modality for long-term monitoring of the disorder should be stressed. A case-control study by Craine et al. [25] using EnteroTach analysis found the sound-to-sound interval (SSI) to be shortest in irritable bowel syndrome (IBS), followed by Crohn’s disease and largest in healthy controls. However, this study lacked specificity as patients with Crohn’s disease having concurrent IBS symptoms were not considered. A study [62] comparing 2-min bowel sound recording in patients with IBD and drug-induced motility with mosapride and senna using EnteroTach analysis found no significant different in the sound-to-sound interval of both groups. The study concluded that the method fails to diagnose hyper-motility conditions and requires a longer recording interval. Therefore, further research is required to establish the diagnostic yield of bowel sounds in this scenario.

### 4.4. Ascites

Ascites [43,44] refers to the collection of fluid in the abdominal cavity that is a sequel to decompensated liver cirrhosis. The third spacing of the fluid causes hypovolemia and alters the hemodynamic status, warranting swift diagnosis. Moderate to severe ascites is diagnosed with a bedside examination, but smaller volumes require imaging modalities. The abdominal ultrasound [63] can study fluid volume greater than 100 mL, but its results are affected by obesity, abdominal mass or distension. Computed tomography scans are an effective diagnostic modality but are not cost-effective. Liatsos et al. [26] studied bowel sound analysis for non-invasive diagnosis of small volume ascites using scatter plots of higher order crossings, which resulted in a significant difference in the bowel sound pattern amongst cases versus healthy controls. However, the small study sample could not determine the sensitivity and specificity of the technique.

### 4.5. Post-Operative Complications and Critical Care

Abdominal surgery increases the risk of complications such as post-operative ileus (POI), and infection and sepsis [27,64]. Post-operative ileus is the decreased intestinal activity due to surgery and anesthesia on bowel motility [13,27]. After surgery, bowel motility is characterized by initial segmental sounds, followed by gradual progression to propulsive sounds that mark the return to normal [13]. Anesthesia used during surgery affects bowel motility [64] and causes an immediate decrease in bowel sounds after surgery with a return to the normal state 3 h later. Kaneshiro et al. [27] used abdominal vibrations and acoustic signals to calculate intestinal rate in patients with post-operative ileus versus normal bowel recovery and found a significantly low intestinal rate in POI cases. Bowel sound analysis can be used for assessing gut activity to guide timely initiation of enteral feeds [65] and administration of purgatives and enema [13] to allow faster recovery from surgery. Additionally, bowel sounds can also be used to measure of severity of post-operative sepsis and guide management strategies. A study [66] noted that gastrointestinal motility decreases with increasing severity of sepsis that was gauged by the level of interleukin-6. Management with oral steroids increased the gastrointestinal motility and proved the treatment to be effective.

Auscultation of bowel sounds in critically ill patients is a valuable tool but has a subjective nature with technical limitations [67]. Although physicians can diagnose ileus by the auscultation of bowel sounds [6], the conventional stethoscope has been unreliable in promptly detecting ileus, with poor sensitivity with low positive predictive value [7,27,65]. Additionally, a noisy environment in the intensive care unit (ICU) makes it even more difficult to auscultate effectively [7]. Bowel sounds may not be a true measure [68] of gastrointestinal function in patients on mechanical ventilation and neuromuscular blocking agents as they swallow little air leading to a decreased intraluminal gas production. A study [69] found bowel sounds to have no association between flatus, bowel activity or tolerance to oral feeds in patients who underwent abdominal surgery and concluded the method was unreliable for determining time to start oral feeds and resolution of postoperative ileus. Similarly, Massey [70] found no association between bowel sounds and return of bowel activity after postoperative ileus, thus doubting the application of this science. Another study also observed some ICU patients with ileus showing the presence of bowel sounds instead of absence [71]. Thus, the discrepancies in literature and technical difficulties encourage the need for further research on pathophysiology and recording technologies [72].

### 4.6. Irritable Bowel Syndrome

Irritable bowel syndrome (IBS) is a chronic functional gastrointestinal disorder [2] characterized by abdominal pain associated with change in stool consistency and frequency, with no structural pathology on endoscopy. IBS is a clinical diagnosis based on patient’s symptoms. Most patients commonly undergo invasive testing with colonoscopies to rule out other disorders before being diagnosed with IBS [9]. The lack of definite testing negatively impacts the affected population causing mental and financial strain [3,73]. Bowel sounds can be used for non-invasive monitoring of gastrointestinal activity in patients with severe diarrhea by recording the vibrations on the surface of the abdomen and processing the signals from the system with a computer [74]. Several studies have tested analysis of bowel sounds to be a potential diagnostic modality for IBS [4,17,25,48,61,73,75]. Craine et al. [17] in a case-control study noted a decreased fasting sound to sound interval (SSI) in IBS cases that was comparable to the decreased post-meal SSI in healthy controls. However, a similar average intensity and frequency of bowel sounds was noted in both groups. Bowel sounds have been extensively studied in IBS patients using computerized auscultation, two-dimensional positional mapping and enterotachogram analysis [25,61,75]. Patients with IBS had a short SSI as compared to healthy volunteers and IBD cases [25]. A significant increase in low frequency sounds was seen in healthy volunteers as compared to functional bowel disorders [75]. Studies [48,73] have found an increased post-meal bowel sounds with a higher density noted in healthy subjects when compared to IBS cases. Upon two-dimensional mapping, the right lower quadrant and mid-upper abdomen were the most active areas of bowel sound production. Further research is needed to identify specific bowel sound characteristics in IBS to formulate a diagnostic modality.

### 4.7. Diabetes Mellitus

Optimal blood glucose regulation is important for normal function of the vital organs [76]. Patients with diabetes mellitus must balance their caloric intake to avoid fluctuations in blood glucose that are affected by physiological factors such as time of food intake, the type of food, exercise, sleep, stress and digestion [29]. Bowel sounds can be studied to understand the post-meal gastrointestinal motility [77] in diabetics and healthy subjects. In diabetic patients, both the sound index (SI) and motility index (MI) decreases, while healthy subjects have an increased SI and MI observed in the gastroduodenal region compared to intestinal region.

An artificial pancreas system [28] is a device comprising a measuring unit that continuously monitors blood glucose to determine the appropriate time and amount of insulin bolus needed. Currently this device fails to measure the effect of dynamic physiological factors on blood glucose [78,79,80]. Mamun and Khandaker et al. [28,29] integrated a bowel sound measuring device within this system to record the digestive state and aid in insulin control. The device detects acoustic vibrations from the bowel for real-time monitoring of food ingestion and intestinal motility, and offers a meal notification feature within the insulin pump to notify the patients of their blood glucose levels [28].

### 4.8. Neurodegenerative Disorders

The dorsal motor nucleus of the vagus nerve [81] forms the parasympathetic nerve supply to the upper gastrointestinal tract mainly the stomach. Neurodegenerative conditions such as Parkinson’s disease, multiple system atrophy and progressive supranuclear palsy damage this nucleus leading to gastroparesis. The slow forward movement of food significantly decreases the bowel sounds in such cases as compared to healthy controls [30]. Assessing bowel motility in these patients allows for timely intervention and prevents further complications.

### 4.9. Neonates

Premature or low birth-weight infants have an immature digestive system and are prone to various gastrointestinal abnormalities [82] such as necrotizing enterocolitis (NEC), vomiting, gastroesophageal reflux, pulmonary aspiration of gastric contents, electrolyte abnormalities, allergies, birth defects, enzyme deficiencies, systemic illnesses, infection, abnormal vascular supply and obstruction. Radiological techniques, although highly specific, delay the time of diagnosis and cannot be used for gastrointestinal monitoring. Hill et al. [82,83] recorded bowel sounds to continuously monitor and relay information to the physician for timely prevention of complications and diagnosis, as well as to determine the appropriate time for enteral nutrition as the premature gut is prone to rejection. A study [84] showed bowel sounds to have high accuracy in diagnosing early stages of NEC development. Tele-diagnostic ability for recording infantile bowel sounds at home using a smartphone and relaying these smartphone data to the clinician is yet another leveraging factor. Pyloric stenosis, an obstructive condition characterized by a hypertrophic gastric outlet has an excellent prognosis when detected early. It is surgically treated by pyloromyotomy, the effectiveness of which can be monitored by recording bowel sounds [31]. Fewer bowel sounds are heard prior to the procedure, due to the obstructive nature of the disease and delayed gastric emptying. The gut sounds reach their normal frequency 48–72 h post-operation. Hence, bowel sounds can be easily used as a reflection of severity of illness, aid in monitoring the post-operative status and help determine time to commence post-operative feeding.

## 5. Auscultation and Recording Technologies

Although auscultation forms an integral part of bedside clinical examination, the use of a stethoscope for listening to the abdominal sounds has limited use. This can be attributed to the poor quality of the recording device and interference by surrounding noise [85,86]. A suction microphone with a crystal inset and phonocardiogram amplifier [16] was used in the 1960s to determine peristalsis in order to diagnose motility disorders. However, the need for simultaneous recording of sound and motility limited the use of this device. Various changes in the structure of stethoscope have been made since the 20th century to improve the quality of auscultation. The primitive stethoscopes with a microphone-based sensor [87,88,89] relied on power supply and was highly sensitive to airborne noise. Using a similar device, real-time monitoring [90] of intestinal motility was obtained from single or multiple bursts pattern of bowel sounds. Soon, non-contact microphones [91,92] were used, but required longer duration of recording making it uncomfortable for the patient. Other modifications that followed were skin adhering stethoscopes [93] and stethoscopes with a diaphragm replaced by a piezoelectric transducer [74]. Bray et al. [94] studied the workings of the transducer device during fasting and post-meals. He noted 500 to 700 Hz epigastric sounds during fasting, a low gastric activity in the inter-digestive state and an increase in the gastric and intestinal sounds post-meals.

Electronic stethoscopes can be used to study conditions of acute abdomen [95] as well as to differentiate patients [96] with small bowel obstruction and postoperative ileus. However, the technique was ineffective due to variability in auditory characteristics across clinicians and surgeons. Although studies [58] show inter-physician agreement in categorizing auscultated bowel sounds into normal and pathological, it cannot be applied clinically due to physician inconvenience. Eventually, all the conventional recording technologies were proved insignificant [97] due to dependence on the operator’s knowledge, interruptions by the surrounding air, need for longer supervision, and poor detection of low amplitude bowel sounds. Bowel sound auscultation was digitally revolutionized with the development of computerized bowel sound detectors coupled to microphone-based sensors. These devices were able to adequately detect subtypes of bowel sounds with accurate start and end points [98]. Single burst (SB), multiple bursts (MB), continuous random sound (CRS) and harmonic sound (HS) patterns were recognized that were previously not detected by any device [98].

The drawbacks of auscultation include a lack of specific guidelines for the area and duration of auscultation. Some scientists state no specific site for bowel sound auscultation [99,100,101] as sounds generated from any location could radiate to the entire abdomen, whereas some [102,103] proposed specific auscultating regions. The advised auscultation duration varies from 30 s to 7 min [102,104,105], preferably prior to palpation [106,107] as it may stimulate peristalsis [99]. However, a recent study [108] found no difference in bowel sounds before and after palpation. An attempt [109] to localize the source of bowel sounds using absorbent and non-absorbent sound propagation models found majority of the sounds [110] in mid-lower and the right lower abdomen. A similar association with the right lower quadrant was revealed by Wang et al. [98] while studying effect of food intake on bowel sounds. They noticed increased number of sounds from this region and explained it by the movement of the ileo-caecal valve upon food consumption [98].

Bowel sound auscultation has transformed multiple folds over the past century, but some studies [96] show its unworthiness to detect bowel pathologies. There is a need of a standardized procedure for auscultation and development of novel technologies that can record and analyze bowel sounds efficiently. Incorporating digital processing of the auscultated sounds could minimize human error and prevent excess recruitment of skilled health care staff. Recent studies [21,22] have developed recording technologies to create a wearable device that is Bluetooth enabled for wireless transmission of data, thus allowing remote and telemedicine healthcare practices. A study by Kutsumi et al. [111] recorded BS using a prototype application on a smartphone and successfully recognized BS using a CNN model. Hence, BS auscultation has a future potential to form a non-invasive diagnosis of various gastrointestinal disorders.

The studies between 1967 and 2022 on this data set have been summarized in Table 1 [21,22,28,42,61,65,83,87,88,89,91,111,112,113,114,115,116,117,118,119,120,121,122,123,124,125,126,127,128,129,130,131,132,133].

## 6. Discussion

Bowel sounds have a promising potential as a non-invasive diagnostic modality and management aids are needed in practice to establish patient-friendly, cost-effective care. We reviewed previous studies to understand how bowel sounds are produced, evaluate the need of bowel sound auscultation or recording in clinical practice, and the future of phonoenterogram in healthcare. Studies explained that bowel sound production are scarce with varied theories. Some studies [12,13,14] link bowel sounds to gut motility, whereas others [39,40] believe it to be due to the transfer of energy between luminal contents. Collectively, the production of bowel sounds could be due to a combination of luminal contents [34], amount of luminal air [33], type of contractions [13], and the myoelectrical activity of the intestine [40]. Physiology-focused studies are required to establish a definite origin.

Despite limited knowledge on the genesis of bowel sounds, phonoenterograms have been applied in various conditions such as intestinal obstruction [57], irritable bowel syndrome [17,25,48,61,75], acute gastrointestinal conditions [24], inflammatory bowel disease [61], diverticular disease [61], bowel polyps [61], postoperative ileus [27], critical care [64], sepsis [66], ascites [63], diabetes mellitus [28,29], neurodegenerative disorders [30], neonatal care [83] and hypertrophic pyloric stenosis [31]. A recent systematic review [19] concluded that computerized analysis of bowel sounds shows promise in the field of diagnostic and prognostic gastroenterology. When integrated with engineering knowledge to create a standardized recording and analysis device this could turn into a powerful technology in the field of gastroenterology.

Auscultation of bowel sounds has been in practice since the time of Hippocrates [12]. This ancient practice was later studied by multiple researchers but had limited usability due to interference of surrounding medium air, with long duration of recordings ultimately leading to erroneous results [71,87,88,89,91,92,97]. Subsequently, the use of procedures such as endoscopy, colonoscopy and manometry increased and are widely employed today. Despite their high accuracy rate, they pose the risk of perforation which can be life-threatening [11]. Thus, there is a need for an inexpensive, non-invasive, patient-friendly alternative for bedside diagnosis of common gastrointestinal conditions.

A phonoenterogram has the potential to revolutionize clinical practice. Research should be focused on building a system that not only records bowel sounds efficiently but also interprets the results accurately. Such a system could eliminate the factor of human error and inter-personal variability involved with the auscultation of bowel sounds. Using this system, a large database for normal and pathological bowel sounds could be created to increase the accuracy of computerized interpretation. This data set can also be used for food evaluation technology, which developing value-added foods based on an individual’s constitution, predisposing conditions and bowel activity [87]. In the future, a digital system capable of recording bowel sounds remotely would be helpful in the monitoring and diagnosis of bed-bound critical patients, and older adults unable to visit the clinic. In addition, a model for self-diagnosis of irritable bowel syndrome could help diagnose this chronic functional motility disorder early and reduce the mental and financial strain on the affected population and the healthcare system.

### Digital Phonoenterography Using Microwave-Based Systems: Future Perspectives

Tomomasa et al. first proposed the relationship between migrating motor complex (MMC) and bowel sounds (BS), suggesting that the sound index synchronizes with the MMC cycle, and BS can potentially be a biomarker for clinical use [40]. However, as the current sensors have various limitations, as discussed above, a novel technique is needed that can effectively measure BS with a phonoenterogram (PEG). Electromagnetic (EM) based sensors have been explored in medicine for continuous vital monitoring. Most of these applications have been used in detecting heart sounds [134]. Microwave energy has recently gained attention, and its applications in healthcare are tremendous, including diagnostic and therapeutic methods [135]. Microwaves are non-ionizing EM waves and are helpful in the development of new treatments and biosensor diagnostics [136].

EM detects audible signals as the reflected radiation from the vibrating object has amplitude modulation representing the vibration. Kumar developed a microwave acoustic detection system to detect vibrating signals by speech through a wall [137]. Researchers have also developed non-contact microwave radar sensors for structural vibration monitoring. With significant advancements and focused radiation beams, microwave technology is used to create auditory radars for vocal signal detection [138]. Lin et al. developed a coherent homodyne demodulator to detect the radar signal reflected from vibrating vocal cords of human subjects. These measured signals are consistent with acoustic signals and have a variety of potential medical applications [139]. Therefore, microwave energy can be used to create wireless sensing applications to detect internal body sounds. Wireless microwave acoustic sensors have been developed and used in various industries, but their application in health remains unexplored [140,141].

Liu. S et al. summarized the theories and applications of electromagnetic acoustic (EMA) techniques in biomedical applications [142]. They found that electromagnetic and acoustic techniques are superior to conventional ultrasound techniques as they have better tolerance to sound speed variation than ultrasonic propagation. Although these studies have shown potential applications, EMA is yet to be applied clinically. Hui et al. [143] demonstrated the UHF microwave technique to retrieve heart sounds. They created a microwave near-field coherent sensor that adapted a radio frequency identification (RFID) tag and compared it to the conventional acoustic stethoscope, which showed similar heart sound content and can be used as a biometric parameter. This can also be used for diagnostic purposes regardless of the ambient noise level [143]. These results suggest the potential design of microwave acoustic PEG sensor for high fidelity data capturing of BS. AI assisted acoustic sensor designs using novel metamaterials offer huge promise for digital phonoenterography using microwave telemetry system.

**Figure 3:**
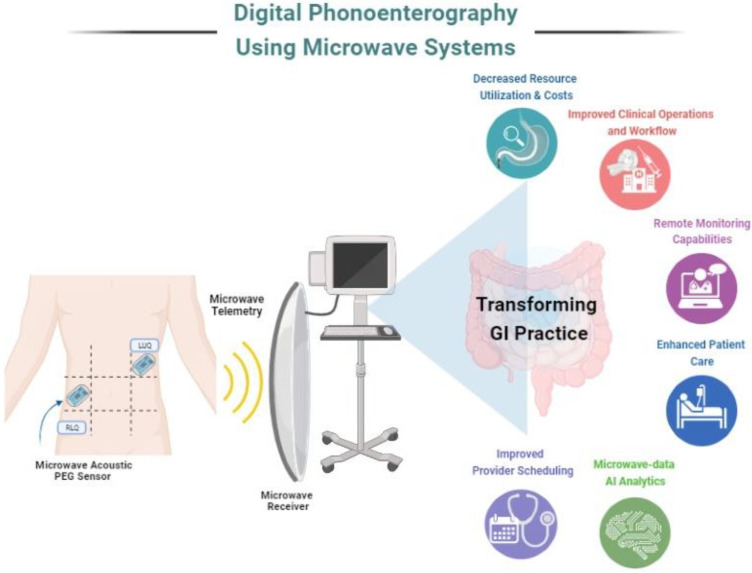
Pictorial representation of digital phonoenterography using microwave systems.

Biomedical telemetry is extensively employed in the ambulatory monitoring of physiological data such as heart rate, blood pressure, oxygen saturation and respiratory rate [144,145,146,147]. The use of microwave energy to monitor vital signs is gaining popularity. Continuous wave radar has been used to monitor heart rate and blood pressure. Various antenna design developments helped improve the use of microwaves in telemetry [148]. Most conventional monitoring systems use inductive transmission for data transfer and device recharge, with problems with high power requirements and biocompatibility. However, high-frequency (~400 MHz) microwave devices with small implantable antennas can serve the same purpose with better battery life and compatibility. Therefore, a microwave telemetry system can complement the digital phonoenterography system design for efficient wireless transmission. Figure 3 [37] depicts an implementation example for digital phonoenterography using microwave systems and its potential impact.

Novel microwave-based acoustic PEG sensors will pave the way for the accurate capturing of bowel sounds. Data transmission using microwave telemetry may employ AI in both PEG data mining and interpretation as well as in the design of a computer-aided decision support system for the accurate diagnosis of GI diseases. Digital phonoenterography assisted with microwave-based systems can positively impact practice operations as well as enhance patient care. With an efficient system in place, healthcare providers can appreciate its impact in terms of reduced resource utilization, lowered operational costs, improved provider scheduling and workflows, remote digital health monitoring as well as an opportunity for data analytics using AI with microwave data to optimize these utilities.

Future research is warranted on the design of novel AI-assisted microwave acoustic sensors specific to the application of interest for digital phonoenterography. Novel AI-assisted metamaterial designs and frequency selective surfaces for microwave acoustic sensors offer huge promise to propel this field. AI-assisted microwave telemetry system design is needed to provide noise free phonoenterography data transmission for reliable diagnosis. Overall, it is evident that the non-invasive diagnosis of GI diseases is warranted, with novel AI-assisted microwave tools that can impact GI practice and patient care. This review provides new insights and directions for practicing digital gastroenterology using a microwave based phonoenterography system.

## 7. Conclusions

Preliminary studies show promising results in the usefulness of bowel sounds in GI practice, though more research is warranted. Research related to the origin of normal bowel sounds as well as the pathophysiology of abnormal bowel sounds is needed to effectively translate acoustic features into clinical practice. The recording and analysis of bowel sounds shows tremendous potential for creating a device that is accessible, patient-friendly, cost-effective and, most importantly, devoid of any risk factors that are associated with radiation or intervention. Microwave-based digital phonoenterography offers a huge opportunity to impact both GI practice as well as patient care. Future research should focus on the design of novel AI-assisted microwave acoustic sensors and telemetry system designs.

## Figures and Tables

**Table 1:** Studies with bowel sounds recording technologies and analytical methods.

Year, Author	Study	Technique	Results & Limitations
1967, Georgoulis [112]	Intestinal sounds classification in post-operative patients.	Capsule microphone → tape recorder → B filter → paper record.	Simple and compound sounds showing interpersonal variation. Requires 48-h long recording.
1988, Radnitz [113]	Biofeedback with bowel sounds for irritable bowel syndrome patients.	Audio-visual bowel sound recording for training patient’s bowel activity.	Reduced mean daily diarrhea reporting, maintained up to 1 year, affected by stress.
1998, Hadjileontiadis [114]	Symmetrical alpha-stable distribution for lung sounds and bowel sounds analysis.	15 to 30 s signal → converter (sampling rate of 2.5 KHz for lung, and 5 KHz for bowel) → WTST-NST and inverse filter.	Contaminated signal- alpha is ~1.5. Denoised signal- alpha decreased significantly.
1994, Sugrue [115]	Computer aided sound analysis system (C.A.S.A.S) in acute abdomen cases vs. healthy controls.	Microphone → analog to digital converter (ADC) → computerized analysis for bowel sounds features	Increased mean sound length and amplitude, and reduced frequency in cases. Patients need to remain still during recording.
1999, Hadjileontiadis [61]	Higher order crossings (HOC) in large bowel disorders vs. healthy controls.	Audioscope → WTST-NST filter → Number of axis crossings (equally spaced points of time) counted → HOC pattern plotted.	Post-polypectomy HOC comparable to control, proving efficacy of the procedure.
2000, Hadjileontiadis [116]	Wavelet based stationary and non-stationary filter (WTST-NST).	Signal divided with wavelet transform (WT) → decomposed into multiple scales with applied power and threshold → filtered with WT coefficient → denoised signal.	Efficiently removed interfering noises and enhanced signal quality.
2001, Ranta [117]	Bowel sound processing (denoising, segmentation and characterization) based on wavelet-based algorithm ^(39)^	Multiple microphones to localize bowel sounds → wavelet coefficients vector with feature extraction for segmentation	Correct interpretation and decontamination of recorded data needed.
2003, Hadjileontiadis [118]	Bowel sound enhancement with reduction of background noise.	Kurtosis-based detector → time domain of explosive bowel sounds → separated from background noise.	Reliable detector for extracting bowel sound peaks.
2003, 2005, Hadjileontiadis [119,120]	To detect explosive lung and bowel sounds in patients with pulmonary and gastrointestinal pathology respectively.	Fractal Dimension (FD) based detector in wavelet transform (WT) domain → detects FD variation and WT coefficients related to lung or bowel sounds.	Low noise susceptibility proved with noise stress test.
2008, Dimoulas [121]	Autonomous intestinal motility analysis for long-term bowel sound monitoring.	Time-frequency features and wavelet parameters in combination with multi-layer perceptron.	Recognition accuracy of 94.84% and 2.19% error in separating interfering noises
2008, Hill [76]	Efficacy of a novel device in NICU patients before and after feeding.	Electronic stethoscope → amplifier → acquisition card → computer → picked up hyperactive bowel sound	Significant background noise not accounted for.
2011, Kim [88]	Modified iterative kurtosis-based detector and estimation algorithms based on regression model of jitter and shimmer.	Piezo-polymer microphone → filtered, digitized, segmented, modified (kurtosis-based algorithm) and characterized (absolute jitter and shimmer method)	Longer colon transit time in delayed bowel motility cases. Small sample size. Lack of technical specifications of the device.
2011, Kim [89]	Back propagation neural network (BPNN) and Artificial neural network (ANN)	Signal modified (kurtosis-based algorithm) and characterized (absolute jitter and shimmer)→ analyzed using BPNN and ANN model.	Longer colon transit time in delayed gastric emptying and spinal cord injury cases. Short sample size and duration of recordings.
2011, Tsai [42]	LabVIEW technique for real-time monitoring of bowel sounds.	Electric condenser microphone attached to a stethoscope → data acquisition interface.	Proved the effectiveness of the digital infinite impulse responses (IIR) filter.
2013, Lin [122]	Higher order statistics based radial basis function network.	A three-layer network with input, hidden and output layers to augment and enhance sound.	Enhancement of bowel sounds during both stationary and non-stationary conditions.
2013, Sakata [87]	Fasting and post meals bowel sounds in healthy volunteers.	Recording device with sensors and built-in amplifiers → computer with WTST-NST filter	Unsynchronized recording of stethoscope and device with conditions not indicative of normal digestive activities.
2014, Spiegel [65]	Bowel sounds in patients with post-operative ileus (POI) vs. those tolerating oral feed.	Real time monitoring using a surveillance biosensor.	Intestinal rate of healthy controls → patients tolerating oral feeds → POI. Failed to isolate coordinated bowel activity.
2015, Mamun [123]	Low power integrated bowel sound measurement system.	Piezoelectric film used as a sensor, amplified, filtered and characterized.	Detected regularly sustained bowel sounds from surrounding noises.
2015, Longfu [124]	Spectral entropy for bowel sound signal identification.	Dynamic weighing threshold and spectral subtraction for detecting and increasing signal to noise ratio (SNR)	Accurate detection of endpoint of bowel sounds in low SNR condition.
2014, Sheu [125]	Higher order crossings-based fractal dimension method in noisy conditions	Recorded bowel sounds → analyzed using higher order crossings.	Superior performance to conventional fractal dimension algorithms.
2015, Yin [126]	Artificial neural network to recognize digestive state.	Extracted bowel sounds → adaptive filtering using 2 reference signals → least mean square algorithm for denoising → threshold detection block	Detected the ongoing digestive state in 3 volunteers.
2016, Mamun [28]	Ultra-low power real time bowel sound detector to measure meal instances in artificial pancreas device.	Piezoelectric sensor → transduced into voltage signal by front end processor → feature extractor identifies bowel sound segment.	Consumes 53microW power from 1V supply in 0.96 mm^2^ area. Suitable for portable devices with 85% accuracy and low false positive rates.
2018, Sato [91]	Non-contact bowel sound analysis after consumption of carbonated water.	Bowel sound segment detection → extraction→ classification → evaluation to detect signal to noise ratio (SNR)	Number of bowel sound segments inversely related to SNR. Accuracy inversely related to post-meals SNR. Small sample size & low sound pressure in stethoscope.
2018, Liu [127]	Mel Frequency Cepstrum Coefficient Feature (MFCC) and Long Short-Term Memory (LSTM) neural network.	Compressed 1 min voice recording→ screened by two doctors for presence or absence of sound signals → further processing and extraction.	Effective results in same environment; decreased sensitivity with noisy signals.
2019, Kolle [128]	Filtering of bowel sounds using multivariate empirical mode decomposition.	Model increases the non-linear components of signals and separates them from other signals.	False events identified and filtered out with easy identification of relevant events. Contamination by artefacts.
2020, Kodani [129]	Long-term bowel sound measurement with elimination of movement-related cloth rubbing noises.	Portable sensor, with the notch, wavelet and low-pass filters → increase focus on bowel sounds and cloth-rubbing noise → separated based on the number of peaks at specific frequency signals.	Effective in differentiating bowel sounds from noise. Difficulty in separating when both overlap.
2020, Zhao [130]	Long-term bowel sound monitoring with Convolutional Neural Network (CNN).	Wearable bowel sound system used for monitoring and CNNs used for segment recognition.	High sensitivity and moderate accuracy for bowel sound monitoring. Time consuming. Noisy-labels present.
2020, Zheng [131]	Convolutional Recurrent Neural Network (CRNN) system-based sound detection.	Gastrointestinal sound set with collection instrument, dataset annotation and distribution, to detect bowel sounds, speech, snoring, cough, rub and groan.	Effective in identifying snore and cough. Weak performance due to low frequency of bowel sound.
2021, Namikawa [132]	Real time bowel sound analysis system for peri-operative monitoring in gastric surgery patients.	Recording equipment and acoustic sensors used to record frequency of bowel sounds.	Frequency of bowel sound was higher in post-gastrectomy cases, with inverse relation to operation time. Small sample size & large-sized equipment.
2021, Ficek [133]	Hybrid convolutional, recursive neural network for bowel sound analysis.	Intestinal sound contact microphone → analyzed using deep neural network.	Efficiently analyzed bowel sound sequences. Lacks wireless technology.
2022, Sitaula [84]	Convolutional Neural Network (CNN) to classify neonatal bowel sounds.	Digital stethoscope recording → computer analysis based on CNN system → refined with Laplace hidden semi-Markov model	Classified bowel sounds into peristaltic and non-peristaltic. Imbalanced data without noise cancellation.
2022, Zhao [22]	Binarized CNN-based BS recognition algorithm with time-domain histogram features for wearable device.	Wearable BS recorder → Gateway via Bluetooth → relayed to cloud servers (wired or wireless)	Algorithm reached 99.92% classification accuracy and very low false alarm rate. Validated by hardware implementation and computation overhead reduction ratio of 58.28 for overall operation.
2022, Wang [21]	Flexible dual-channel digital auscultation patch with active noise reduction for long-term BS monitoring.	Digital auscultation patch (two channels for BS and one channel for ambient noise) → transmitted via Bluetooth → computer processing with adaptive filtering for active noise reduction, feature extraction and source localization → BS analysis created with intelligent systems.	Flexible, soft, light patch can easily bend to maintain conformal attachment on the abdomen. Wireless wearable device is suitable for long term monitoring. Noise reducing algorithm is useful in noisy clinical environments.
2022, Kutsumi [111]	Prototype smartphone application to record BS using built-in microphone with automatic analyzation of BS.	BS recorded with built-in microphone of Apple iPhone 7 using the BS recording application. Annotated BS segments were analyzed using CNN and LSTM models.	The CNN model was superior and recognized BS with moderate accuracy (88.9%) with data recorded from a smartphone.

## Data Availability

The review was based on publicly available academic literature databases.
